# Antioxidant and Anti-Inflammatory Effects of Herbal Formula SC-E3 in Lipopolysaccharide-Stimulated RAW 264.7 Macrophages

**DOI:** 10.1155/2017/1725246

**Published:** 2017-10-15

**Authors:** Soo Chil Lee, Young-Won Kwon, Ju-Yeon Park, Sung Yun Park, Ju-Hee Lee, Sun-Dong Park

**Affiliations:** College of Korean Medicine, Dongguk University, Goyang 10326, Republic of Korea

## Abstract

SC-E3 is a novel herbal formula composed of five oriental medicinal herbs that are used to treat a wide range of inflammatory diseases in Korean traditional medicine. In this study, we sought to determine the effects of SC-E3 on free radical generation and inflammatory response in lipopolysaccharide- (LPS-) treated RAW 264.7 macrophages and the molecular mechanism involved. The ethanol extract of SC-E3 showed good free radical scavenging activity and inhibited LPS-induced reactive oxygen species generation. SC-E3 significantly inhibited the production of the LPS-induced inflammatory mediators, nitric oxide and prostaglandin E_2_, by suppressing the expressions of inducible nitric oxide synthase and cyclooxygenase-2, respectively. SC-E3 also prevented the secretion of the proinflammatory cytokines, IL-1*β*, TNF-*α*, and IL-6, and inhibited LPS-induced NF-*κ*B activation and the mitogen-activated protein kinase (MAPK) pathway. Furthermore, SC-E3 induced the expression of heme oxygenase-1 (HO-1) by promoting the nuclear translocation and transactivation of Nrf2. Taken together, these results suggest that SC-E3 has potent antioxidant and anti-inflammatory effects and that these effects are due to the inhibitions of NF-*κ*B and MAPK and the induction of Nrf2-mediated HO-1 expression in macrophages. These findings provide scientific evidence supporting the potential use of SC-E3 for the treatment and prevention of various inflammatory diseases.

## 1. Introduction

Interest in diseases and health problems and desires for a better quality of life are rising along with an increase in life expectancy. Since most disease is associated with the disruption of homeostasis and chronic inflammation, oxidative stress and inflammation have become major health issues. Oxidative stress is defined as an imbalance between the production of reactive oxygen species (ROS) and the capacity of cellular antioxidant defenses [1]. Excessive ROS generation damages cellular macromolecules, including proteins, carbohydrates, lipids, and nucleic acids, dysregulates cellular signaling events, and ultimately leads to the pathogeneses and progressions of inflammatory diseases [2]. Although inflammation and oxidative stress are often by-products of normal cellular processes, excessive oxidative stress and chronic inflammation can cause chronic diseases, such as diabetes, cancer, degenerative diseases, and obesity, and accelerate the aging process [3]. Therefore, it is important that oxidative stress and inflammation be adequately controlled to prevent the progressions of chronic diseases.

Macrophages are key modulator and effector cells in immune response and play critical roles in the initiation, maintenance, and resolution of inflammation [4]. When exposed to inflammatory stimuli, macrophages become activated and then increase the productions of inflammatory mediators, such as nitric oxide (NO) and prostaglandin E_2_ (PGE_2_), and of inflammatory cytokines, such as tumor necrosis factor- (TNF-) *α*, interleukin- (IL-) 1, and IL-6 [4, 5]. These inflammatory responses have been extensively studied in RAW 264.7 macrophages stimulated by lipopolysaccharide (LPS, an endotoxin obtained from gram-negative bacteria) [6].

Traditional Korean herbal medicines have long been used to treat various inflammatory diseases. Over the past decades, the usages of herbs or traditional Korean herbal medicines that are complementary and/or alternative medicines to the management of inflammation have increased because of concerns about the adverse side effects of nonsteroidal anti-inflammatory drugs [7]. Accordingly, we initiated this study to identify traditional Korean herbal medicines that might be useful for the prevention or treatment of inflammatory conditions. Sanghanron (Shang Han Lun) and Geumgweyoryak (Jin Gui Yao Lue) are ancient Chinese medical books written by Jang Jung Kyung (Zhang Zhong-jing) and constitute the basis of traditional Korean herbal prescriptions [8, 9]. We designed three novel anti-inflammatory herbal formulas called SC-E1, SC-E2, and SC-E3, based on prescriptions for treating inflammation in the Sanghanron and Geumgweyoryak, and each herbal formula was derived based on the daily doses of five herbal medicines (Table 1). Preliminary* in vitro* screening showed that SC-E3, which contains five medicinal herbs (Bupleuri Radix, Coptidis Rhizoma, Gardeniae Fructus, Rhei Rhizoma, and Puerariae Radix), exhibited greatest free radical scavenging ability and most inhibited NO production. Therefore, in the present study, we evaluated the antioxidant and anti-inflammatory effects of SC-E3 in LPS-stimulated RAW 264.7 macrophages and sought to identify the mechanisms responsible for its effects.

## 2. Materials and Methods

### 2.1. Chemicals and Reagents

Dulbecco's Modified Eagle's Medium was purchased from Welgene (Gyeongsan, Korea) and fetal bovine serum (FBS) from Gibco BRL (Gaithersburg, MD, USA). ELISA kits for IL-1*β*, TNF-*α*, and IL-6 were obtained from Ab Frontier (Seoul, Korea) and PGE_2_ was purchased from R&D Systems (Minneapolis, MN, USA). Primary antibodies, that is, anti-HO-1, anti-COX-2, anti-iNOS, anti-p-I*κ*B-*α*, anti-p-NF-*κ*B (p65), and anti-Nrf2 and secondary antibodies were purchased from Santa Cruz Biotechnology (Santa Cruz, CA, USA). Dimethyl sulfoxide (DMSO) was purchased from Junsei Chemical Co. (Tokyo, Japan), and LPS (*E. coli* 055:B5), geniposide, puerarin, 3-(4,5-dimethylthiazol-2-yl)-2,5-diphenyl-tetrazolium bromide (MTT), Griess reagent, 4,6-diamidino-2-phenylindole (DAPI), and other reagents were purchased from Sigma-Aldrich (St. Louis, MO, USA).

### 2.2. Isolation and Structural Identification of SC-E3

SC-E3 was formulated from five oriental medicinal herbs, that is, Bupleuri Radix (voucher specimen number: DUMCKM2015-107), Coptidis Rhizoma (voucher specimen number: DUMCKM2015-083), Gardeniae Fructus (voucher specimen number: DUMCKM2015-069), Rhei Rhizoma (voucher specimen number: DUMCKM2015-017), and Puerariae Radix (voucher specimen number: DUMCKM2015-001). All were purchased as dried herbs from Omniherb (Daegu, Korea) in accord with the good manufacturing practices (GMP) procedures certified by the Korea Food and Drug Administration (KFDA) and authenticated by Professor Sun-Dong Park (Department of Prescriptions, College of Korean Medicine, Dongguk University). Voucher specimens were deposited at the College of Korean Medicine, Dongguk University.

Briefly, a mixture of dried Bupleuri Radix, Coptidis Rhizoma, Gardeniae Fructus, Rhei Rhizoma, and Puerariae Radix (100 g; weight ratios 3 : 1 : 3 : 1 : 3) was macerated in 800 mL of 70% ethanol, stirred for 24 h at room temperature (RT), and filtered twice through an 8 *μ*m Whatman filter paper. After rotary evaporation at 40~45°C, the concentrate was lyophilized using a freeze dryer (EYELA, Japan). The yield of the SC-E3 extract (dried powder) was 15.2% by weight with respect to the dried starting materials.

### 2.3. High-Performance Liquid Chromatography (HPLC)

SC-E3 was analyzed by a Dionex Ultimate 3000 HPLC system (Thermo Fisher Scientific, Waltham, MA, USA) equipped with a binary solvent delivery pump, a vacuum degasser, an autosampler, a temperature controlled column oven (30°C), and a diode array spectrophotometric detector (DAD). Geniposide and puerarin (Sigma-Aldrich) were used as standards. Separations were performed using a VDSpher EC-C18 column (4.6 mm × 250 mm, 5 *μ*m, VDSoptilab, Germany). The mobile phase consisted of 0.3% trifluoroacetic acid (A) and acetonitrile (B), and gradient elution was performed as follows: 10% B for 0-1 min, 10–50% B for 1–25 min, 90% B for 25–35 min, and 90–10% B for 35–40 min. The flow rate and injection volume were 0.8 mL/min and 10 *μ*L, respectively. The assays were monitored at 240 nm and all data were acquired and processed using Chromeleon 6.8 software.

### 2.4. DPPH Radical Scavenging Activity Assay

A 2,2-Diphenyl-1-picrylhydrazyl (DPPH) assay was conducted using a slight modification of the method described by Gyamfi et al. [10]. Briefly, beforehand DPPH solution (0.1 mM) was prepared by dissolving 3.94 mg DPPH in 100 mL of ethanol. Various concentrations of SC-E3 (50–500 *μ*g/mL) were incubated in 50 mM Tris-HCl (pH 7.4) containing 0.1 mM DPPH for 30 min in the dark. The control was prepared as above but without SC-E3. Absorbances (Abs.) were measured at 517 nm using a microplate reader. Scavenging activity was defined as the percentage of DPPH radicals scavenged and was calculated using the following equation:

(1) Scavenging  effect%=control  Abs.−sample  Abs.control  Abs.×100.

### 2.5. Superoxide Anion Free Radical Scavenging Activity Assay

Scavenging activities for the superoxide anion free radical were assessed as previously described with slight modification [11]. Briefly, samples of SC-E3 (0, 50, 100, 300, or 500 *μ*g/mL) were added to a reaction solution containing 30 *μ*L of 30 mM EDTA (pH 7.4), 10 *μ*L of 30 mM hypoxanthine in 50 mM NaOH, and 200 *μ*L of 1.42 mM nitro blue tetrazolium (NBT). This solution was preincubated at RT for 3 min and then 10 *μ*L of 1 U/mL xanthine oxidase was added followed by 50 mM of phosphate buffer (pH 7.4) to bring the volume up to 300 *μ*L. This solution was incubated at RT for 20 min and then absorbance was measured at 560 nm.

### 2.6. Total Polyphenol Content

The total phenolic content of SC-E3 was determined using the Folin-Ciocalteu colorimetric method as described by Ainsworth and Gillespie with slight modification [12]. Briefly, 40 *μ*L of SC-E3 was added to 200 *μ*L of Folin-Ciocalteu reagent (Sigma-Aldrich) in 1,160 *μ*L of distilled water and mixed thoroughly. The mixture was incubated for 3 min at RT and to this 600 *μ*L of 2% sodium carbonate was added. After 2 h of incubation in dark, the mixture was aliquoted into a 96-well plate and absorbance was measured at 765 nm using a microplate reader. Gallic acid was used as standard and total phenolic content was expressed as mg of gallic acid equivalents (GAE) per gram of SC-E3.

### 2.7. Total Flavonoid Content

The total flavonoid content of SC-E3 was estimated using an aluminium trichloride colorimetric method using catechin as the reference compound [13]. SC-E3 (100 *μ*L) was mixed with 400 *μ*L of distilled water and added to 30 *μ*L of 5% sodium nitride (NaNO_2_). After 6 min, 30 *μ*L of 10% aluminium trichloride (AlCl_3_) was added to the mixture and incubated for 5 min, followed by the addition of 200 *μ*L of 1 M NaOH. The final volume of the mixture was adjusted to 1 mL with distilled water and incubated for 15 min, and then absorbance was measured at 510 nm using a microplate reader. Total flavonoid content was calculated using a catechin standard curve and results were expressed as mg of catechin equivalents (CE) per gram of SC-E3.

### 2.8. Cell Culture

RAW 264.7 macrophages were purchased from the American Type Culture Collection (Rockville, MD, USA) and cultured in Dulbecco's Modified Eagle's Medium, supplemented with 10% FBS and 1% penicillin-streptomycin in a humidified 5% CO_2_ incubator at 37°C.

### 2.9. Cell Viability

The effect of SC-E3 on cell viability was evaluated using a MTT colorimetric assay. Briefly, cells were suspended in 96-well plates at 1 × 10^4^ cells/well and and treated with different concentrations of SC-E3 for 24 h. Cells were then treated with MTT solution (2 mg/mL) for 3 h. After removing supernatants, the formazan produced was dissolved in DMSO, and absorbance was measured at 540 nm using a microplate reader (Genios, Tecan, Austria).

### 2.10. Reactive Oxygen Species (ROS) Assessments

A fluorescent dichlorofluorescein diacetate (DCFH-DA) assay was used to access intracellular ROS concentrations. Murine macrophages were seeded on a 96-well black plate at 1 × 10^5^ cells/mL, and incubated with LPS (1 *μ*g/mL) in the presence or absence of SC-E3 (50, 100, 300, or 500 *μ*g/mL). After removing medium, cells were treated with 10 *μ*M DCFH-DA in phosphate-buffered saline (PBS) for 30 min at 37°C. Fluorescence was measured at excitation and emission wavelengths of 480 nm and 530 nm, respectively, using a fluorescence microplate reader (Spectra Gemini, Molecular Devices).

### 2.11. Nitrite Assay

RAW 264.7 macrophages were pretreated with various concentrations (50–500 *μ*g/mL) of SC-E3 for 1 h and then stimulated or not for 18 h with LPS (1 *μ*g/mL). Aliquots (100 *μ*L) of supernatants were reacted with equal volumes of Griess reagent [1% sulfanilamide, 0.1% N-(1-naphthyl)-ethylenediamine dihydrochloride, 2.5% phosphoric acid] at RT for 10 min, and nitrite concentrations were assessed by measuring absorbance at 540 nm with a microplate reader.

### 2.12. Enzyme-Linked Immunosorbent Assay (ELISA)

RAW 264.7 macrophages were preincubated with various concentrations (50–500 *μ*g/mL) of SC-E3 for 1 h and then stimulated for 18 h with LPS. Supernatants were harvested, and cytokine contents including those of IL-1*β*, TNF-*α*, and IL-6 were assessed using ELISA kits (Ab Frontier), according to the manufacturer's instructions. PGE_2_ levels were measured using a PGE_2_ parameter assay kit (R&D Systems), according to the manufacturer's instructions.

### 2.13. Nuclear and Cytosolic Fractionation

RAW 264.7 macrophages treated with or without SC-E3 were harvested and washed with PBS. Nuclear and cytoplasmic fraction of cells was conducted using NE-PER™ nuclear and cytoplasmic extraction kit (Thermo Scientific, Rockford, IL, USA) according to the manufacturer's instructions. The nuclear and cytoplasmic extracts were stored at −80°C until required. Protein concentrations in extracts were quantified using a bicinchoninic acid (BCA) protein assay kit (Thermo Scientific).

### 2.14. Western Blot Analysis

RAW 264.7 macrophages were extracted with RIPA lysis buffer containing phosphatase and protease inhibitor cocktail (GenDEPOT, Barker, TX, USA), and total protein concentrations were then determined using a BCA protein assay kit (Thermo Scientific). Equal amounts of total proteins were separated by 10% SDS-PAGE and transferred onto polyvinylidene difluoride membranes, which were then blocked in 5% skim milk for 2 h at RT and incubated overnight at 4°C with primary antibodies against p38, iNOS, COX-2, ERK1/2, JNK, Nrf2, Lamin B, HO-1, *β*-actin, and the phosphorylated forms of p38, NF-*κ*B, I*κ*B-*α*, ERK1/2, and JNK. Membranes were then rinsed and incubated with secondary antibodies conjugated with horseradish peroxidase for 2 h at RT. After rinsing, bands were visualized using ECL prime solution (Amersham Bioscience, Buckinghamshire, UK). Blots were quantified by densitometry using scientific imaging software (ImageJ 1.42; NIH, Bethesda, MD, USA) after normalizing versus *β*-actin, which was used a loading control.

### 2.15. Immunofluorescence Microscopy

To detect the nuclear translocations of NF-*κ*B and Nrf2, RAW 264.7 cells were cultured directly on glass cover slips in 6-well plates and treated with 300 *μ*g/mL SC-E3 in the presence or absence of LPS. Briefly, cells were fixed with methanol for 10 min, permeabilized in PBS containing 1% Triton X-100 for 10 min, incubated with NF-*κ*B p65 or Nrf2 antibody (1 : 200) in PBS overnight at 4°C, and labelled with fluorescein isothiocyanate- (FITC-) conjugated goat anti-rabbit IgG (1 : 1000, Invitrogen) for 1 h and DAPI (Sigma-Aldrich) for 5 min. After mounting coverslips on glass slides using ProLong® Gold Antifade Reagent (Thermo Scientific), fluorescence images were captured using an Olympus BX50 fluorescence microscope (Olympus Optical, Tokyo, Japan).

### 2.16. Statistical Analysis

Results are presented as the means ± standard deviations (SDs) of at least three independent experiments. Statistical significance was determined by one-way ANOVA followed by Tukey's multiple comparison test using GraphPad Prism software (GraphPad Software Inc., San Diego, CA, USA). Differences were considered statistically significant when *p* values were <0.05.

## 3. Results

### 3.1. Screening of the Effects of Novel Herbal Prescriptions

**Figure 1:**
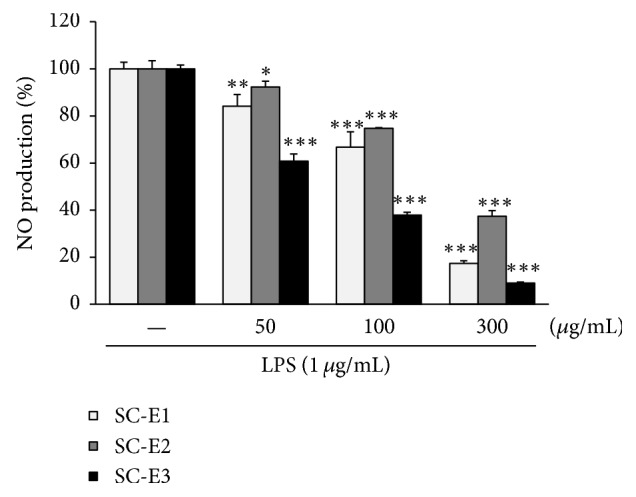
Effects of the three herbal formulas (SC-E1, SC-E2, and SC-E3) on the production of NO in LPS-stimulated RAW 264.7 macrophages. Cells were pretreated with various concentrations (50–300 *μ*g/mL) of each formula for 1 h and then stimulated with LPS (1 *μ*g/mL) for 18 h. NO production was determined using Griess reagent. (Significant versus LPS treatment, ^*∗*^*p* < 0.05, ^*∗∗*^*p* < 0.01, and ^*∗∗∗*^*p* < 0.001.)

To find new antioxidant or anti-inflammatory prescription candidates, we first evaluated the free radical scavenging activities of SC-E1, SC-E2, and SC-E3 extracts. All three extracts exhibited significant, dose-dependent DPPH radical scavenging activity (Supplementary Figure  1(a) in Supplementary Material available online at https://doi.org/10.1155/2017/1725246). The free radical scavenging activities of the extracts in descending order were SC-E3 > SC-E1 > SC-E2. The scavenging effects of the three extracts for the superoxide anion free radical were measured, and SC-E3 was found to have the greatest scavenging activity (Supplementary Figure  1(b)). The three extracts were then screened for their inhibitory effects on NO production in LPS-stimulated RAW 264.7 macrophages. As shown in Figure 1, all extracts significantly and dose-dependently suppressed NO production, and SC-E3 had the most potent effect with an IC_50_ value of 73.6 *μ*g/mL, followed by SC-E1 (IC_50_ = 167.8 *μ*g/mL) and SC-E2 (IC_50_ = 232.4 *μ*g/mL). Based on these results, SC-E3 was selected for further study.

### 3.2. The Antioxidant and Anti-Inflammatory Effects of SC-E3 on RAW 264.7 Macrophages

**Figure 2:**
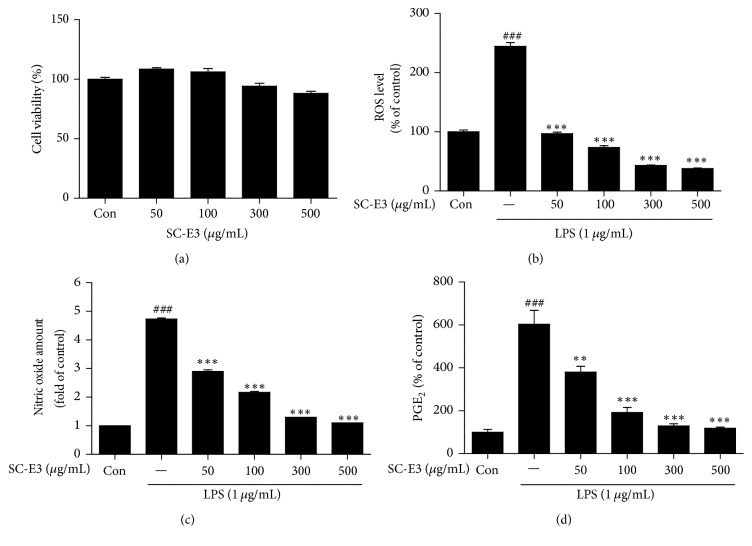
Effects of SC-E3 on ROS generation and on the productions of inflammatory mediators and cytokines in LPS-stimulated RAW 264.7 macrophages. (a) Effects of SC-E3 on viability. Cell viability was determined by MTT assay. Cells were treated with various concentrations of SC-E3 extract (50, 100, 300, or 500 *μ*g/mL) for 24 h. Values were expressed as percentages of the nontreated control. (b) Effect of SC-E3 on ROS generation. Fold increases in intracellular ROS versus nontreated control were determined by measuring DCF fluorescence intensities. (c) Effects of SC-E3 on LPS-induced NO production. Cells were stimulated with 1 *μ*g/mL of LPS, in the absence or presence of various concentrations (50, 100, 300, or 500 *μ*g/mL) of SC-E3 for 18 h. Nitrite production was measured using Griess reagent. (d) Effects of SC-E3 on LPS-induced PGE_2_ production. (Significant versus the nontreated control, ^###^*p* < 0.001, significant versus LPS treatment, ^*∗∗*^*p* < 0.01 and ^*∗∗∗*^*p* < 0.001.)

**Figure 3:**
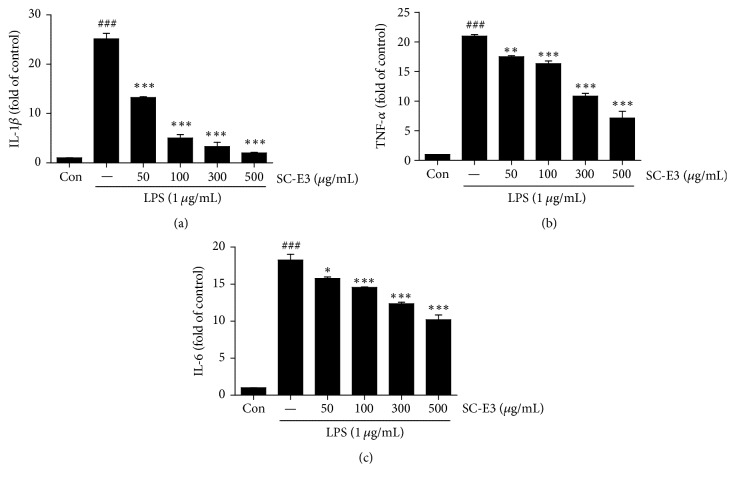
Effects of SC-E3 on LPS-induced proinflammatory cytokine production. Extracellular levels of IL-1*β* (a), TNF-*α* (b), and IL-6 (c) were measured using ELISA kits. (Significant versus the nontreated control, ^###^*p* < 0.001, significant versus LPS treatment, ^*∗*^*p* < 0.05, ^*∗∗*^*p* < 0.01, and ^*∗∗∗*^*p* < 0.001.)

The cytotoxicity of SC-E3 on RAW 264.7 cells was evaluated using a MTT assay. The results showed that SC-E3 at concentrations up to 500 *μ*g/mL had no toxic effect on RAW 264.7 cells (Figure 2(a)). The antioxidant activity of SC-E3 was investigated in RAW 264.7 cells treated with LPS. As shown in Figure 2(b), LPS markedly increased intracellular ROS levels in RAW 264.7 cells versus nontreated controls. However, pretreating cells with various concentrations of SC-E3 (50–500 *μ*g/mL) significantly and dose-dependently reduced LPS-induced ROS generation. In particular, at a concentration of 50 *μ*g/mL SC-E3 suppressed ROS production to the control level. To assess the antioxidant effect of SC-E3 further, its total phenolic and total flavonoid contents were estimated using gallic acid and catechin calibration curves, respectively. The results showed that total phenolic and flavonoid contents of SC-E3 were 11.53 ± 0.61 and 7.04 ± 0.07 mg/g, respectively. Next, we examined the anti-inflammatory effect of SC-E3 by investigating the levels of the proinflammatory mediators NO and PGE_2_ and of the proinflammatory cytokines IL-1*β*, TNF-*α*, and IL-6 in LPS-stimulated RAW 264.7 macrophages. As shown in Figures 2(c) and 2(d), pretreatment with SC-E3 dose-dependently suppressed the LPS-induced productions of NO and PGE_2_. In particular, SC-E3 at 300 or 500 *μ*g/mL inhibited these productions to an extent similar to that of the nontreated control. In addition, pretreatment with SC-E3 significantly reduced LPS-induced increases in the levels of IL-1*β*, TNF-*α*, and IL-6 (Figure 3).

### 3.3. The Inhibitory Effect of SC-E3 on the Expressions of iNOS and COX-2 in LPS-Stimulated RAW 264.7 Macrophages

**Figure 4:**
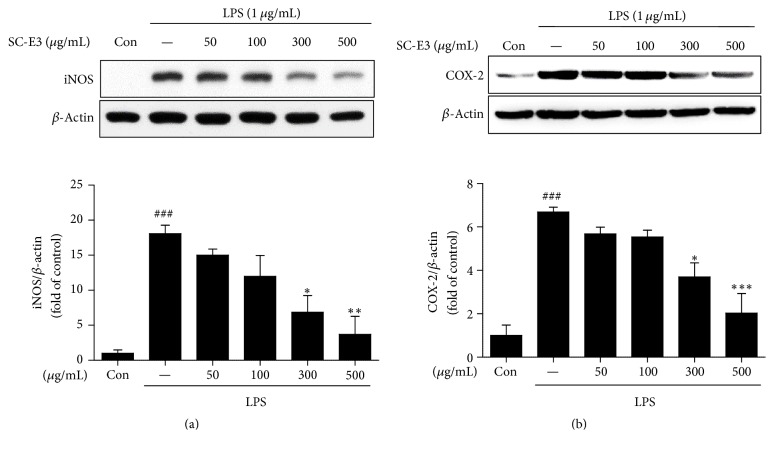
Effects of SC-E3 on the LPS-induced expressions of iNOS and COX-2 proteins in RAW 264.7 macrophages. Protein expressions of iNOS and COX-2 in RAW 264.7 macrophages incubated with different concentrations of SC-E3 (50, 100, 300, or 500 *μ*g/mL) with or without LPS (1 *μ*g/mL) for 24 h were assessed by western blot. The results showed that SC-E3 reduced the protein levels of iNOS (a) and COX-2 (b). (Significant versus nontreated control, ^###^*p* < 0.001, versus LPS treatment, ^*∗*^*p* < 0.05, ^*∗∗*^*p* < 0.01, and ^*∗∗∗*^*p* < 0.001.)

We also evaluated the effects of SC-E3 on the protein levels of iNOS and COX-2, which are involved in the productions of NO and PGE_2_, respectively, in LPS-stimulated RAW 264.7 macrophages. As shown in Figures 4(a) and 4(b), LPS increased the expressions of iNOS and COX-2 in macrophages and these augmentations were reduced dose-dependently by pretreating SC-E3, particularly at SC-E3 concentrations of 300 or 500 *μ*g/mL.

### 3.4. Effect of SC-E3 on Activation of the MAPK Signaling Pathway in LPS-Stimulated RAW 264.7 Macrophages

**Figure 5:**
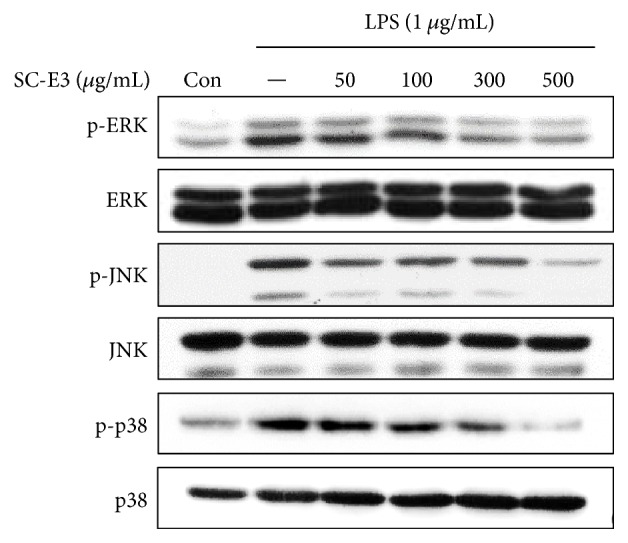
Effects of SC-E3 on MAPK pathway activation in LPS-stimulated RAW 264.7 macrophages. Cells were pretreated with different concentrations of SC-E3 (50, 100, 300, or 500 *μ*g/mL) for 12 h and then stimulated with LPS (1 *μ*g/mL) for 1 h. Western blotting was performed with antibodies for p-ERK, ERK, p-JNK, JNK, p-p38, and p38.

To understand the molecular mechanisms responsible for the anti-inflammatory effects of SC-E3, we examined whether SC-E3 affected MAPK signaling pathways in LPS-stimulated RAW 264.7 macrophages by western blot. ERK1/2, JNK, and p38 (the main kinases of the MAPK pathway) were phosphorylated by LPS stimulation and SC-E3 pretreatment dose-dependently reduced phosphorylation of these kinases, without altering their total form (Figure 5).

### 3.5. Effect of SC-E3 on NF-*κ*B Activation in LPS-Stimulated RAW 264.7 Macrophages

**Figure 6:**
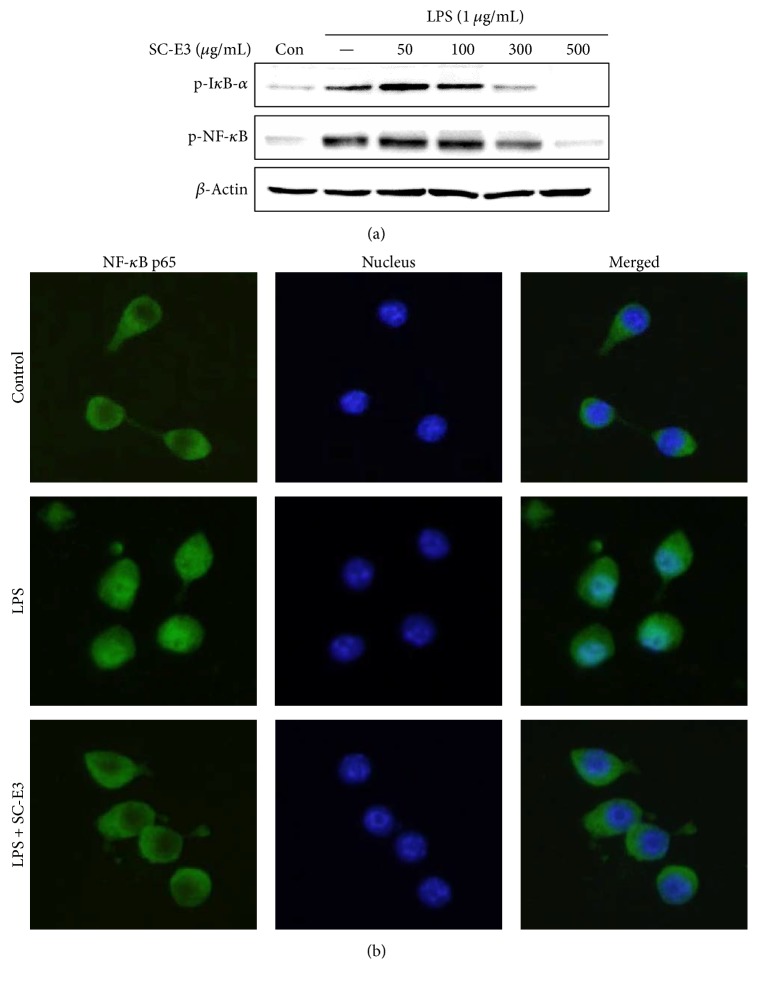
Effects of SC-E3 on NF-*κ*B pathway activation by LPS. RAW 264.7 cells were pretreated with SC-E3 (300 *μ*g/mL) for 18 h and then stimulated with LPS (1 *μ*g/mL) for 1 h. (a) Cell lysates were subjected to western blot analysis to determine the protein levels of p-NF-*κ*B and p-I*κ*B-*α*. (b) The nuclear translocation of NF-*κ*B was observed by immunofluorescence microscopy.

NF-*κ*B activation plays an important role in the regulations and transcriptions of many genes that act as mediators of inflammatory response [14]. To determine whether SC-E3 influences the activation of NF-*κ*B signaling in LPS-stimulated RAW 264.7 macrophages, cells were pretreated with SC-E3 for 18 h and then treated with LPS (1 *μ*g/mL) for 1 h. As shown in Figure 6(a), western blotting showed that the phosphorylation of I*κ*B-*α* and NF-*κ*B was increased after LPS stimulation, and this change was inhibited by pretreating SC-E3. In addition, we also confirmed that pretreatment with SC-E3 at 300 *μ*g/mL suppressed the nuclear translocation of NF-*κ*B under LPS-induced inflammatory condition by fluorescence immunostaining (Figure 6(b)).

### 3.6. Effect of SC-E3 on HO-1 Expression and Nrf2 Activation in RAW 264.7 Macrophages

**Figure 7:**
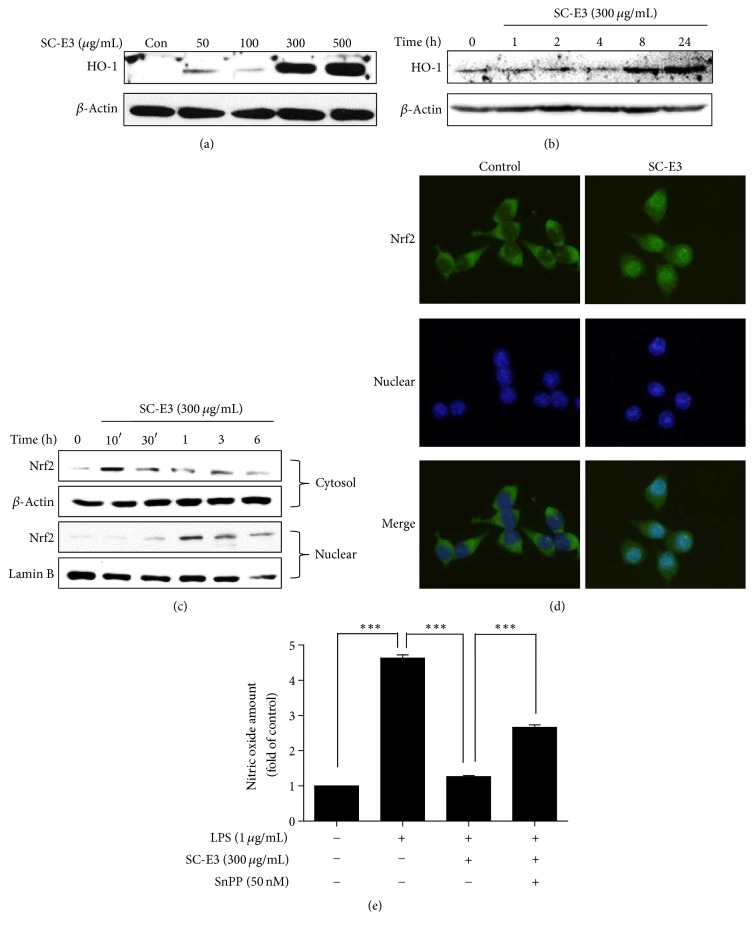
Effects of SC-E3 on the Nrf2/HO-1 signaling pathway in RAW 264.7 macrophages. (a) Induction of HO-1 by SC-E3. Cells were treated with different concentrations of SC-E3 (50, 100, 300, or 500 *μ*g/mL) for 18 h. (b) Cells were treated with 300 *μ*g/mL SC-E3 for the indicated times. (c) Nuclear accumulation of Nrf2 by SC-E3. Nrf2 was immunoblotted in the nuclear fractions of cells treated with 300 *μ*g/mL of SC-E3 for the indicated times. (d) Immunofluorescence images of the nuclear translocation of Nrf2 induced by SC-E3. RAW 264.7 cells were treated with 300 *μ*g/mL of SC-E3 for 3 h. (e) Blocking of the inhibitory effect of SC-E3 on LPS-induced NO production by SnPP (an HO-1 inhibitor). RAW 264.7 cells were pretreated with SC-E3 (300 *μ*g/mL) for 1 h in the presence or absence of SnPP (50 nM, 30 min) and then stimulated with LPS (1 *μ*g/mL) for 18 h. ^*∗∗∗*^*p* < 0.001.

To determine whether the antioxidant and anti-inflammatory effects of SC-E3 are mediated by HO-1 induction, we analyzed HO-1 expression in RAW 264.7 macrophages by western blot. As shown in Figure 7(a), treatment with SC-E3 for 18 h increased HO-1 protein levels in a dose-dependent manner, and this was particularly evident at SC-E3 concentrations of 300 and 500 *μ*g/mL. At a concentration of 300 *μ*g/mL, HO-1 expression increased with time and this increased expression was sustained for 8 h to 24 h after treating cells with SC-E3 (Figure 7(b)). To investigate the molecular mechanism responsible for this upregulation of HO-1 by SC-E3, we assessed the nuclear translocation of Nrf2 in RAW 264.7 macrophages by western blot. As shown in Figure 7(c), nuclear Nrf2 levels were increased by SC-E3 and peaked after 1 h of SC-E3 treatment. Consistent with these results, fluorescence immunostaining showed that treatment with 300 *μ*g/mL SC-E3 led to the translocation of Nrf2 from the cytoplasm into the nucleus (Figure 7(d)). To demonstrate a direct link between HO-1 protein expression and LPS-induced NO production, we examined the effect of SC-E3 on LPS-induced NO production in the presence of SnPP (a HO-1 inhibitor). Inhibition of HO-1 induction by SnPP increased nitrite levels in SC-E3 pretreated macrophages under LPS-induced inflammatory conditions (Figure 7(e)), but SnPP did not fully reverse the inhibitory effects of SC-E3. These results suggest that the anti-inflammatory effect of SC-E3 is mediated, at least in part, by HO-1 induction through Nrf2 translocation.

### 3.7. Identification of Compounds from SC-E3 Extract

**Figure 8:**
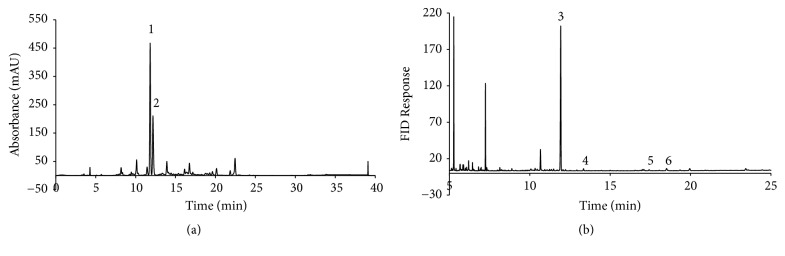
Identification of compounds from the ethanolic extract of SC-E3. (a) HPLC chromatogram of SC-E3 extract. The two compounds, geniposide and puerarin, were selected as marker compounds for quality control. The retention times of geniposide and puerarin were 12.183 and 11.830 min, respectively. (b) Representative GC-MS chromatogram for the analysis of fatty acids in SC-E3. Peaks: 1: puerarin, 2: geniposide, 3: pentadecanoic acid, 4: palmitic acid, 5: oleic acid, and 6: linoleic acid.

For quantitative HPLC analysis, we selected geniposide from Gardeniae Fructus and puerarin from Puerariae Radix and Bupleuri Radix as controls. The concentrations of geniposide and puerarin in SC-E3 were determined using calibration curves prepared using the geniposide and puerarin standards. Calibration curves of geniposide and puerarin showed good linearity with respect to concentration (correlation coefficients (*r*^2^) ≥ 0.9996). The retention times of geniposide and puerarin were 12.173 and 11.823 min, respectively, and the amounts of geniposide and puerarin in SC-E3 extract were 88.9 mg/g and 57.1 mg/g, respectively (Figure 8(a)). Furthermore, GC-MS was applied to identify the compositions of the fatty acids in SC-E3 extract and pentadecanoic acid, palmitic acid, linoleic acid, and oleic acid were detected (Figure 8(b)).

## 4. Discussion

The present study was undertaken to identify new prescriptions for the prevention or treatment of inflammatory conditions based on traditional Korean herbal medicines. These efforts resulted in the development of a novel herbal formula called SC-E3, which is comprised of the following five medicinal herbs: Bupleuri Radix, Coptidis Rhizoma, Gardeniae Fructus, Rhei Rhizoma, and Puerariae Radix. SC-E3 exhibited potent antioxidant and anti-inflammatory effects in RAW 264.7 macrophages and the underlying molecular mechanism responsible was found to involve the inhibitions of NF-*κ*B and MAPK and Nrf2-mediated HO-1 induction.

Prolonged exposure to imbalances in homeostasis can result in a number of diseases. Western medicine generally focuses on diseased parts of the body, while oriental medicine seeks to identify and address the fundamental causes of diseases. The basic principle of traditional oriental medicine is to restore balance and harmony within the body and between individuals and their environments [15]. The use of traditional medicine and of complementary and alternative medicines has increased worldwide over the past decades and its use is supported by empirical evidence on safety and efficacy obtained over thousands of years [16]. In Korea, traditional herbal medicines are commonly in traditional Korean medicine and many formulas (prescriptions) have been developed involving combinations of herbal medicines. Components of these formulas are believed to act synergistically to complement beneficial effects and to neutralize the toxic or adverse effects of individual constituent herbs [17]. These formulas are based on traditional wisdom and experience, and their beneficial effects have been validated by scientific studies [17]. Recently, Kim et al. demonstrated the utility of the “Kun-Shin-Choa-Sa (Jun Chen Zuo Shi) theory,” according to which multiple components of herbal medicines act synergistically by affecting multiple targets [18]. Based on prescriptions describing treatments for inflammation in Sanghanron, which addresses diseases caused by cold factors, and in Geumgweyoryak, which concerns the treatment of miscellaneous diseases, we designed SC-E3 using partly Kun-Shin-Choa-Sa theory.

Of the five herbs constituting SC-E3, Bupleuri Radix, the dried roots of* Bupleurum falcatum* L. (Umbelliferae), is one of the most commonly used crude drugs as a febrifuge in China, Japan, and Korea [19]. Puerariae Radix, the dried root of* Pueraria lobata* Ohwi (Leguminosae), has been traditionally used to relieve fever and dysentery and has been reported to have antioxidant and anti-inflammatory effects [20, 21]. Coptidis Rhizoma,* Coptidis japonica* Makino (Ranunculaceae) is commonly used to treat dermatological disorders in oriental medicine. The anti-inflammatory effects of Coptidis Rhizoma, Gardeniae Fructus (*Gardenia jasminoides* Ellis, Rubiaceae), and Rhei Rhizoma (*Rheum palmatum* L., Polygonaceae) have been previously studied in RAW 264.7 cells [22–24]. Furthermore, a mixture of Coptidis Rhizoma and Rhei Rhizoma was shown to exert its antioxidant and anti-inflammatory effects by regulating NF-*κ*B mediated inflammation in acute reflux esophagitis-induced rats [25].

The present study described the antioxidant and anti-inflammation activities of SC-E3 in LPS-stimulated RAW 264.7 murine macrophages. LPS is one of the most potent innate immune-activating stimuli and induces the productions of cytokines and inflammatory mediators, such as NO and PGE_2_ in macrophages [26]. RAW 264.7 cells have been widely used in studies on macrophage cellular physiology because they are easily cultured, grow rapidly, and phenotypically resemble primary macrophages [27]. Therefore, LPS-stimulated RAW 264.7 macrophages provide a good model for anti-inflammatory drug screening and for subsequently investigating effects on the signal pathways responsible for proinflammatory enzyme induction and for the production of proinflammatory cytokines [28]. In the present study, we found that SC-E3 suppressed iNOS and COX-2 protein levels and LPS-induced ROS generation and thereby suppressed the production of iNOS-derived NO and COX-2-derived PGE_2_ and the secretion of proinflammatory cytokines, that is, IL-1*β*, TNF-*α*, and IL-6.

We tried to unearth the mechanism underlying the anti-inflammatory effect of SC-E3 by focusing on intracellular signaling pathways, such as the MAPK, NF-*κ*B, and Nrf2/HO-1 pathways. MAPKs are a family of serine/threonine protein kinases that mediate fundamental biological processes and cellular responses to external stress signals [29]. LPS activates receptors by binding Toll-like receptor 4, triggers the MAPK pathway, and thus, activates NF-*κ*B [29]. The NF-*κ*B pathway has long been considered a prototypical proinflammatory signaling pathway, because of the role of NF-*κ*B in the expressions of proinflammatory genes, such as cytokines, chemokines, and adhesion molecules [30]. Our results show that SC-E3 inhibited the phosphorylation of MAPKs (i.e., ERK, JNK, and p38 MAPK), I*κ*B-*α*, and NF-*κ*B by LPS stimulation. The nuclear translocation of Nrf2 is associated with the expressions of antioxidant proteins that protect against oxidative damage induced by inflammation [31]. Furthermore, HO-1, a gene targeted by Nrf2 in macrophages, is considered to have anti-inflammatory roles [32, 33]. Moreover, studies have reported that various natural herb extracts attenuate LPS-induced inflammatory responses by modulating Nrf2-mediated HO-1 induction and NF-*κ*B pathways [34–36]. Consistent with these studies, our results also showed that SC-E3 regulates HO-1 induction via Nrf2 activation in macrophages. We also confirmed that treatment with SnPP (a HO-1 inhibitor) blocked the suppression of LPS-induced NO production by SC-E3. These results demonstrate that the anti-inflammatory effects of SC-E3 are mediated by a multitargeting mechanism that involves the inhibitions of MAPK and NF-*κ*B and Nrf2-mediated HO-1 induction in macrophages.

Since SC-E3 is composed of five herbal medicines, it may exhibit a wide range of effects due to the combined effect of various ingredients. Geniposide and puerarin were identified and quantified as the major constituents in the ethanolic extract of SC-E3 by HPLC. These compounds have been reported to exhibit considerable antioxidant and anti-inflammatory activities in various studies [37–44]. Phenolic and flavonoid compounds have also been reported to exhibit antioxidant, anticancer, and anti-inflammatory activities [45–48]. In the present study, the total phenolic and total flavonoid contents in the crude extract from SC-E3 were determined using calibration curves of gallic acid and catechin. Our results suggest that polyphenols and flavonoids might be the contributors for the antioxidant and anti-inflammatory activity of SC-E3 at least in part.

## 5. Conclusion

Taken together, the present study shows that the developed herbal formula SC-E3 has potent antioxidant and anti-inflammatory effects on LPS-induced RAW 264.7 macrophages. Our results suggest that the beneficial effects of SC-E3 may be due to its ability to inhibit the generation of excess ROS and the production of proinflammatory mediators and cytokines by suppressing the activation of MAPK and NF-*κ*B and by enhancing Nrf2-mediated HO-1 induction. Overall, our findings suggest SC-E3 may be considered a new prescription candidate for the treatment of inflammatory diseases. Further studies including preclinical studies are necessary to determine the clinical usefulness of SC-E3.

## Supplementary Material

Supplementary Figure 1: The free radical scavenging activities of the three herbal formulas. (a) DPPH radical scavenging activity. (b) Superoxide anion radical scavenging activity.

## Figures and Tables

**Table 1:** The compositions of the three herbal formulas examined.

Latin name	Scientific name (family name)	Ratio
*SC-E1*		
Gypsum Fibrosum	Gypsum	16
Puerariae Radix	*Pueraria lobata *Ohwi (Leguminosae)	6
Gardeniae Fructus	*Gardenia jasminoides *Ellis (Rubiaceae)	6
Glycyrrhizae Radix et Rhizoma	*Glycyrrhiza uralensis* Fischer (Leguminosae)	2
Platycodi Radix	*Platycodon grandiflorum* A. De Candolle (Campanulaceae)	3

*SC-E2*		
Bupleuri Radix	*Bupleurum falcatum* Linne (Umbelliferae)	6
Coptidis Rhizoma	*Coptidis japonica *Makino (Ranunculaceae)	2
Gardeniae Fructus	*Gardenia jasminoides *Ellis (Rubiaceae)	6
Glycyrrhizae Radix et Rhizoma	*Glycyrrhiza uralensis* Fischer (Leguminosae)	2
Platycodi Radix	*Platycodon grandiflorum* A. De Candolle (Campanulaceae)	3

*SC-E3*		
Bupleuri Radix	*Bupleurum falcatum* Linne (Umbelliferae)	3
Coptidis Rhizoma	*Coptidis japonica *Makino (Ranunculaceae)	1
Gardeniae Fructus	*Gardenia jasminoides *Ellis (Rubiaceae)	3
Rhei Rhizoma	*Rheum palmatum* Linne (Polygonaceae)	1
Puerariae Radix	*Pueraria lobata *Ohwi (Leguminosae)	3

## Abbreviations

ANOVA: Analysis of variance
COX-2: Cyclooxygenase-2
DAPI: 4,6-Diamidino-2-phenylindole
DCFH-DA: 2′,7′-Dichlorofluorescein-diacetate
DPPH: 2,2-Diphenyl-1-picrylhydrazyl
ELISA: Enzyme-linked immunosorbent assay
FBS: Fetal bovine serum
FITC: Fluorescein isothiocyanate
HO-1: Heme oxygenase 1
HPLC: High-performance liquid chromatography
HRP: Horseradish peroxidase
iNOS: Inducible nitric oxide synthase
IL-1*β*: Interleukin-1*β*
IL-6: Interleukin-6
LPS: Lipopolysaccharide
MAPK: Mitogen-activated protein kinase
MTT: 3-(4,5-Dimethylthiazol-2-yl)-2,5-diphenyl-tetrazolium bromide
NO: Nitric oxide
Nrf2: Nuclear factor erythroid 2-related factor 2
NF-*κ*B: Nuclear factor-kappa B
PBS: Phosphate-buffered saline
PGE_2_: Prostaglandin E_2_
ROS: Reactive oxygen species
RT: Room temperature
TNF-*α*: Tumor necrosis factor-*α*
SD: Standard deviation.
