# Supplementary figures and images for: Antioxidant and Anti-Inflammatory Effects of Herbal Formula SC-E3 in Lipopolysaccharide-Stimulated RAW 264.7 Macrophages
Source: Evid Based Complement Alternat Med. 2017 Oct 15;2017:1725246. doi: 10.1155/2017/1725246 (PMC5662831; doi:10.1155/2017/1725246)

(a)

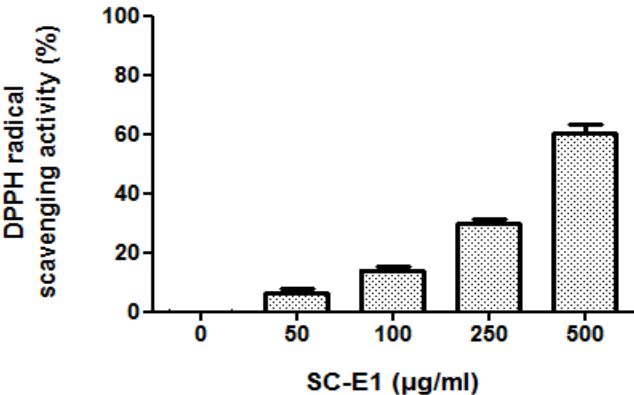

(b)

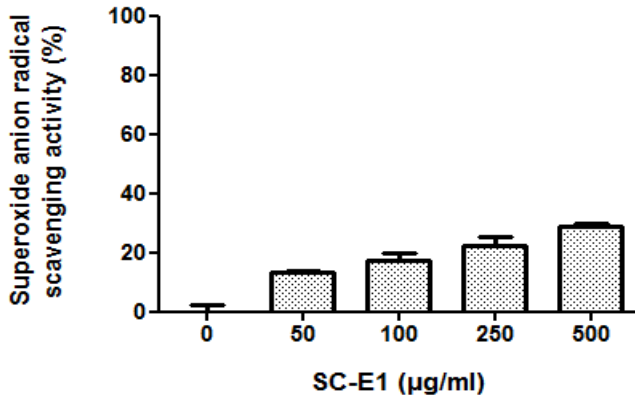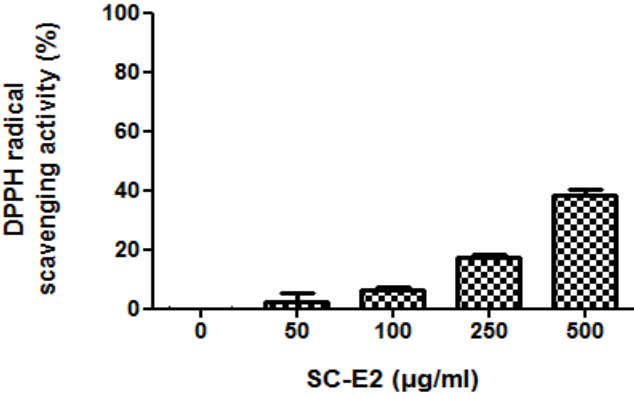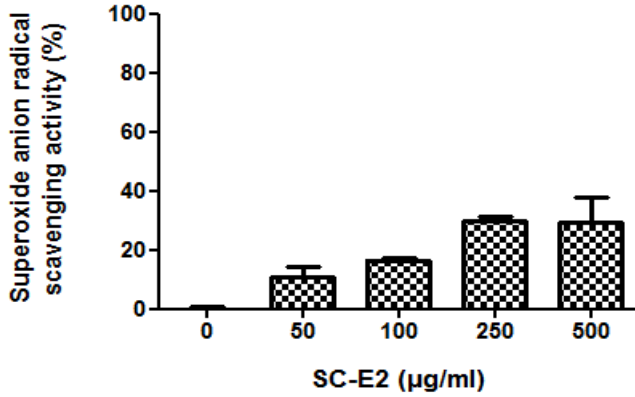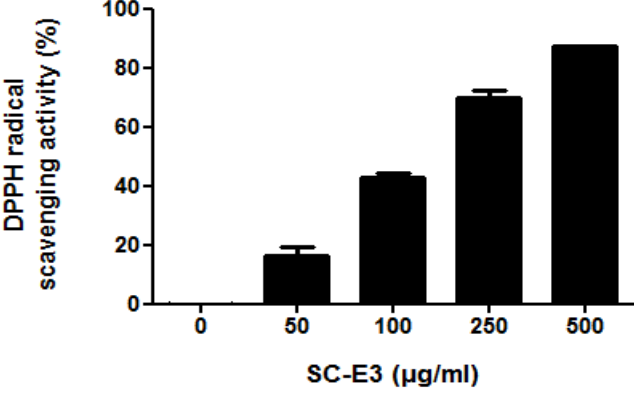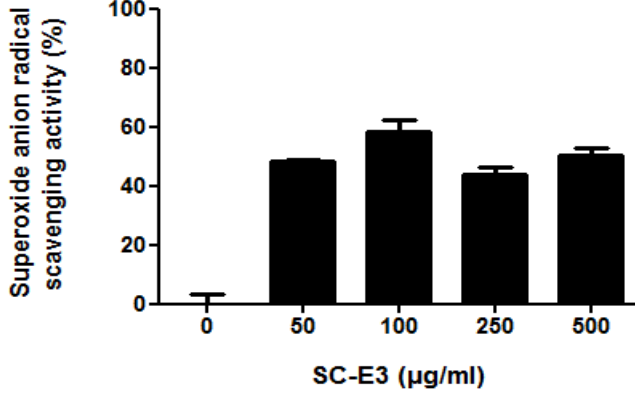

Supplementary FIGURE 1

Supplement: Supplementary file 1 — Supplementary Figure 1: The free radical scavenging activities of the three herbal formulas. (a) DPPH radical scavenging activity. (b) Superoxide anion radical scavenging activity. [file 1725246.f1.pdf]
